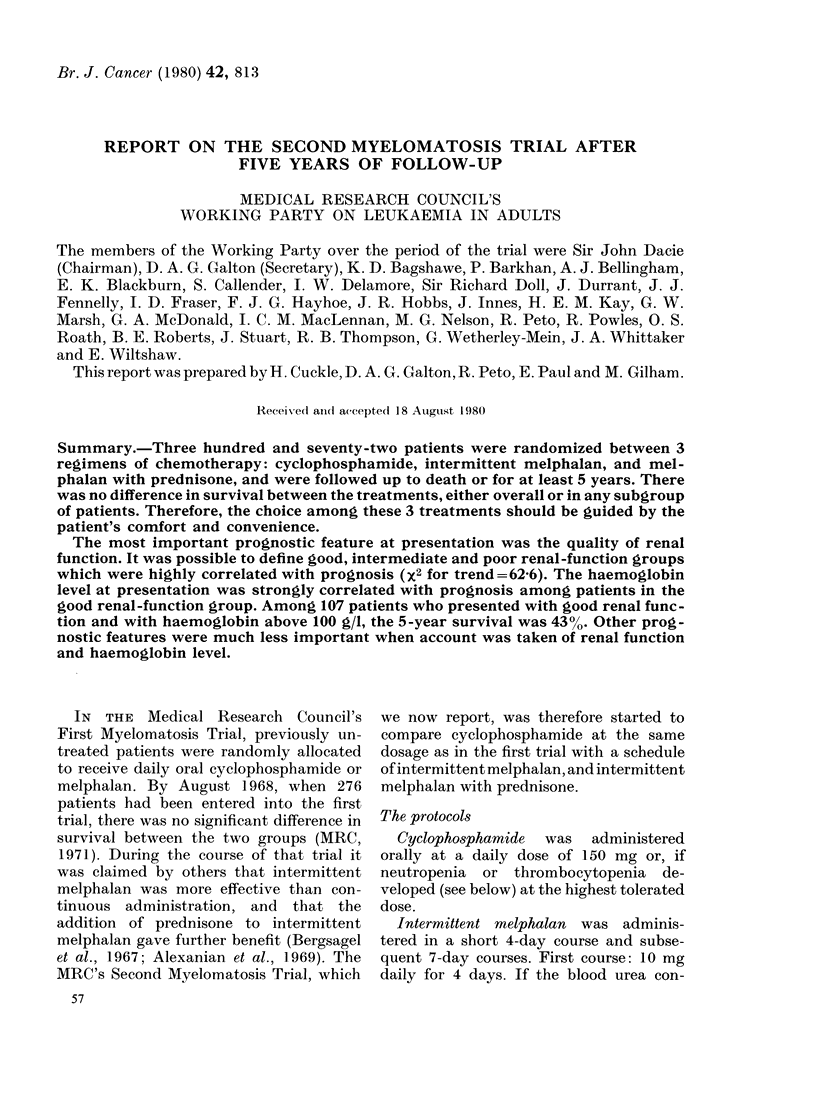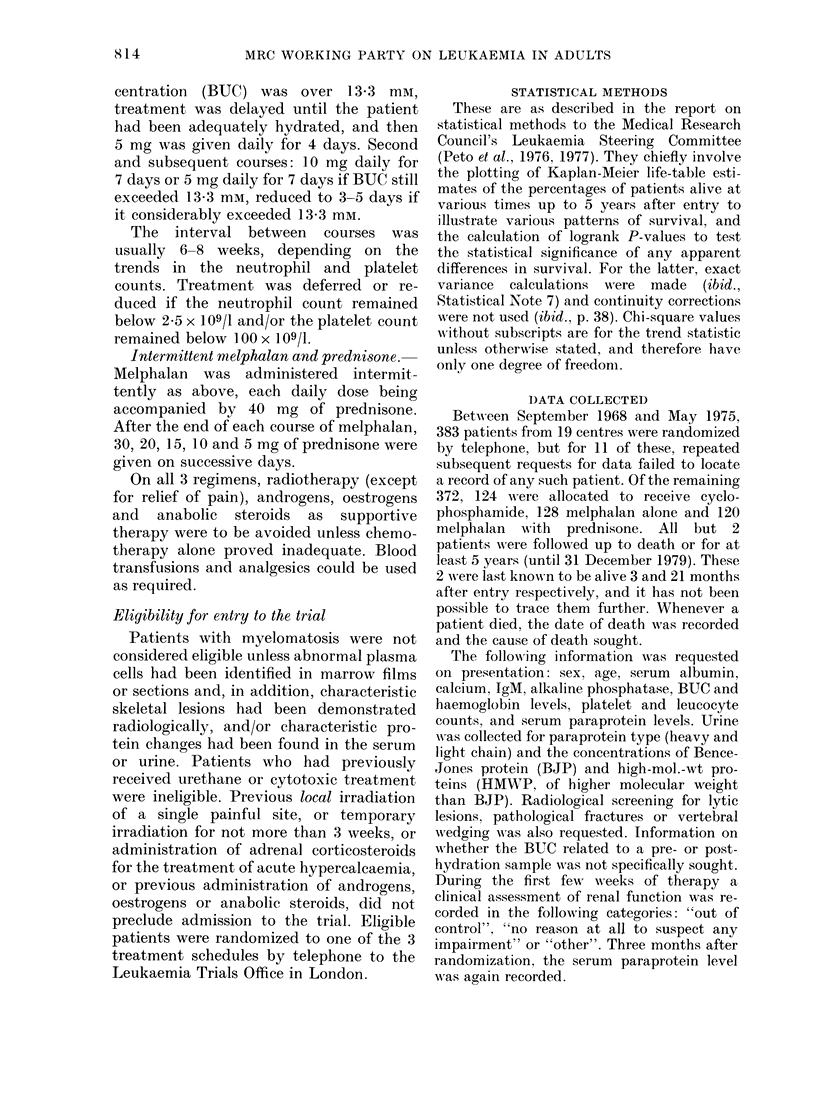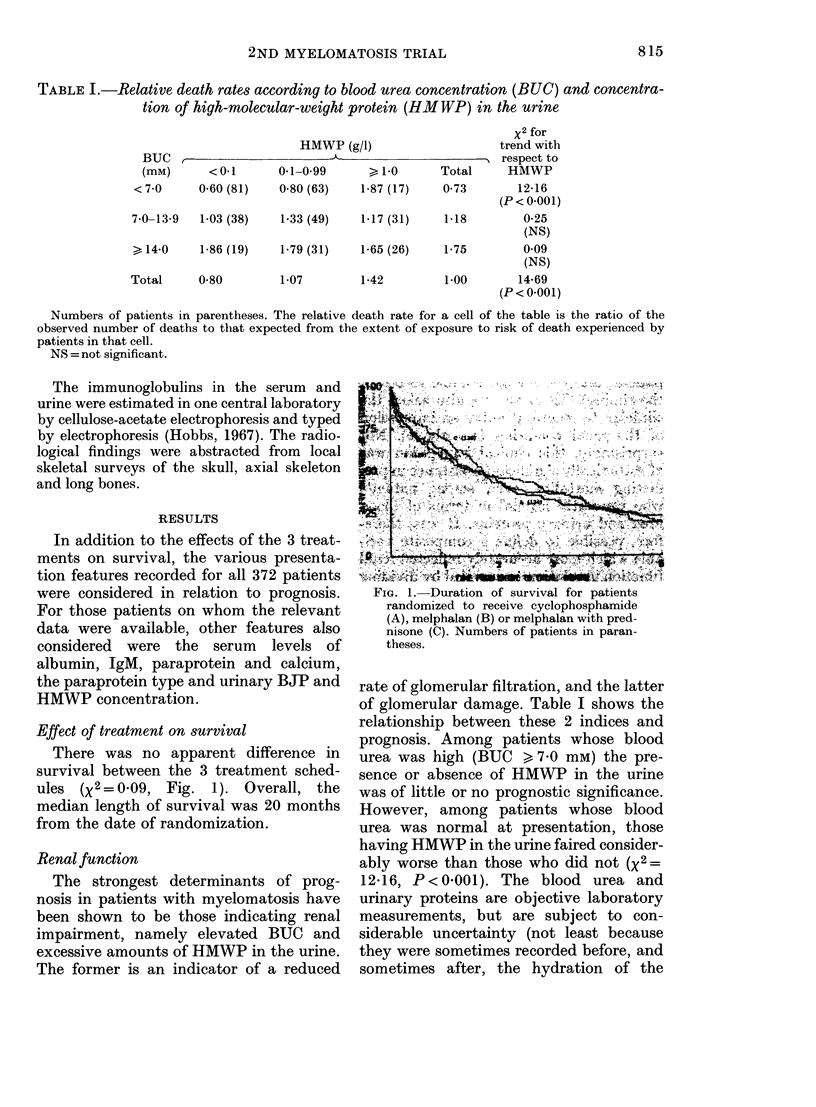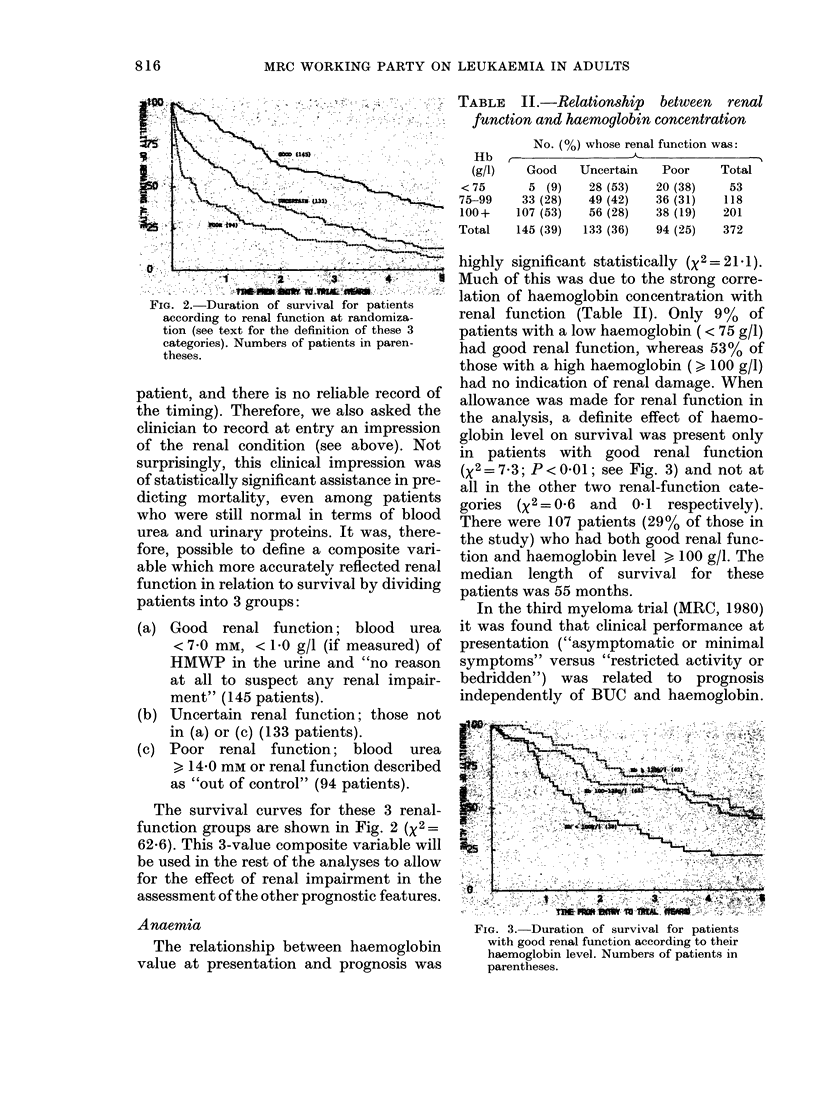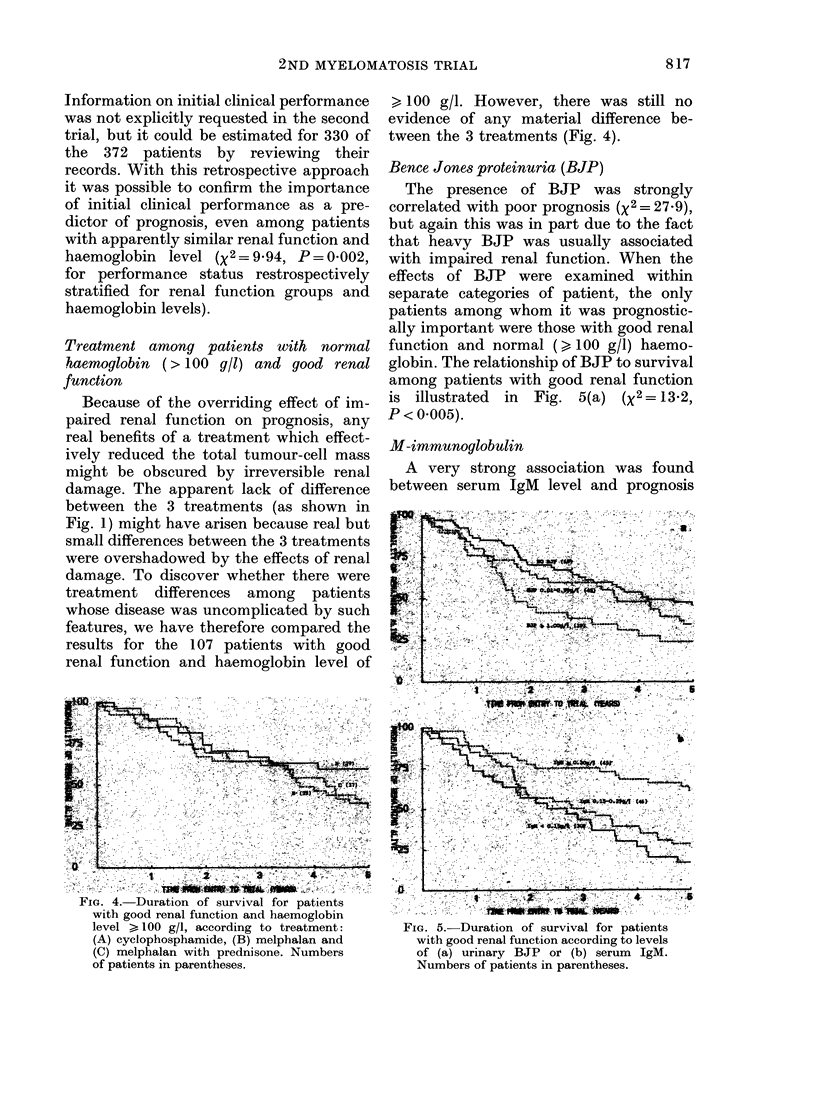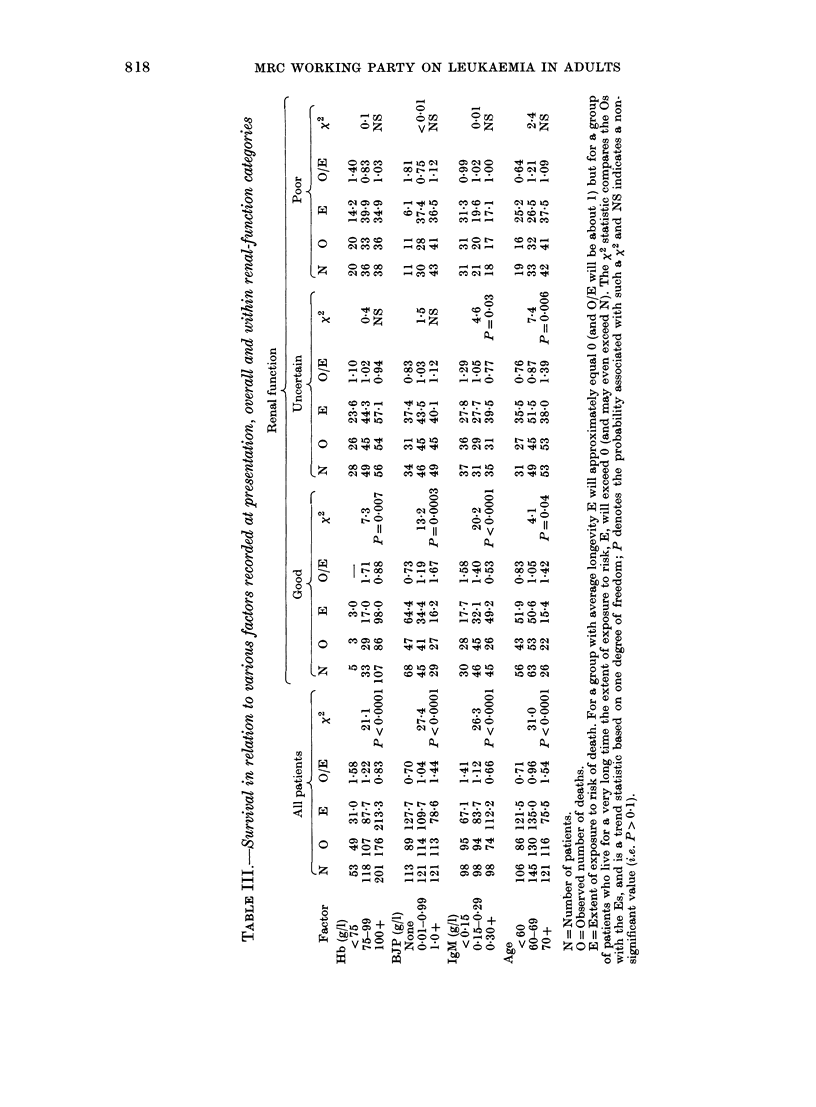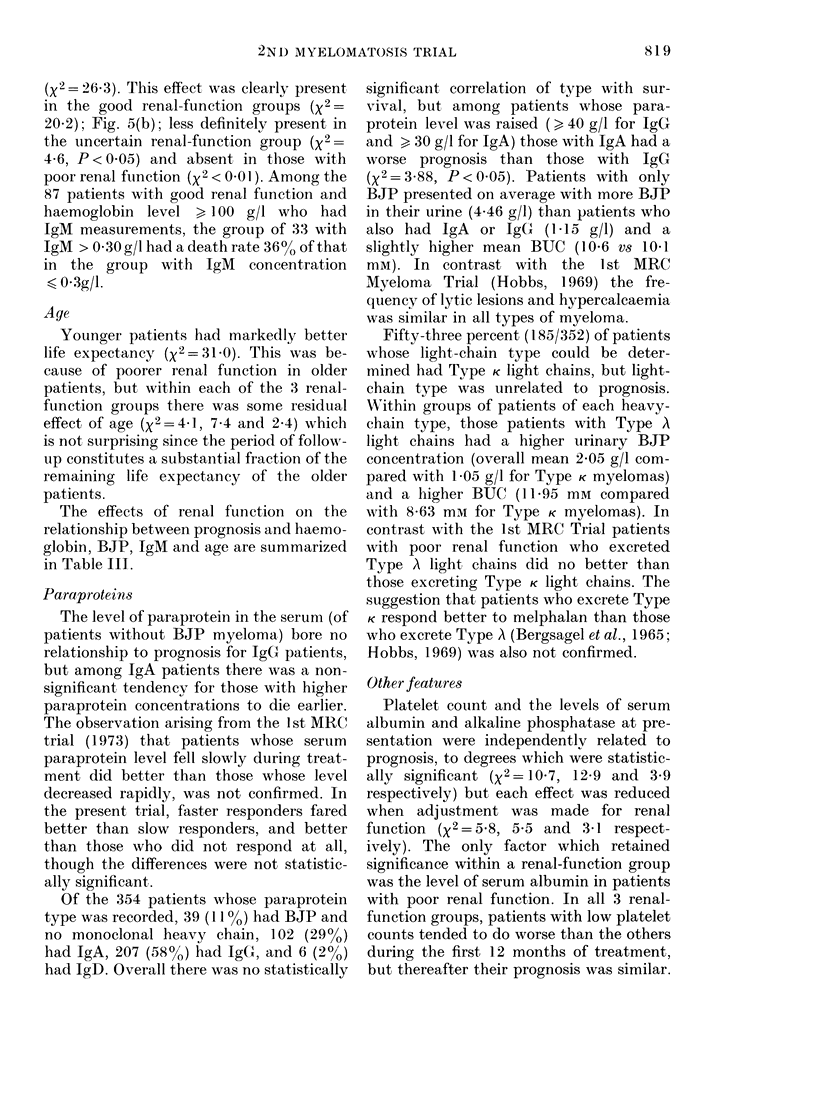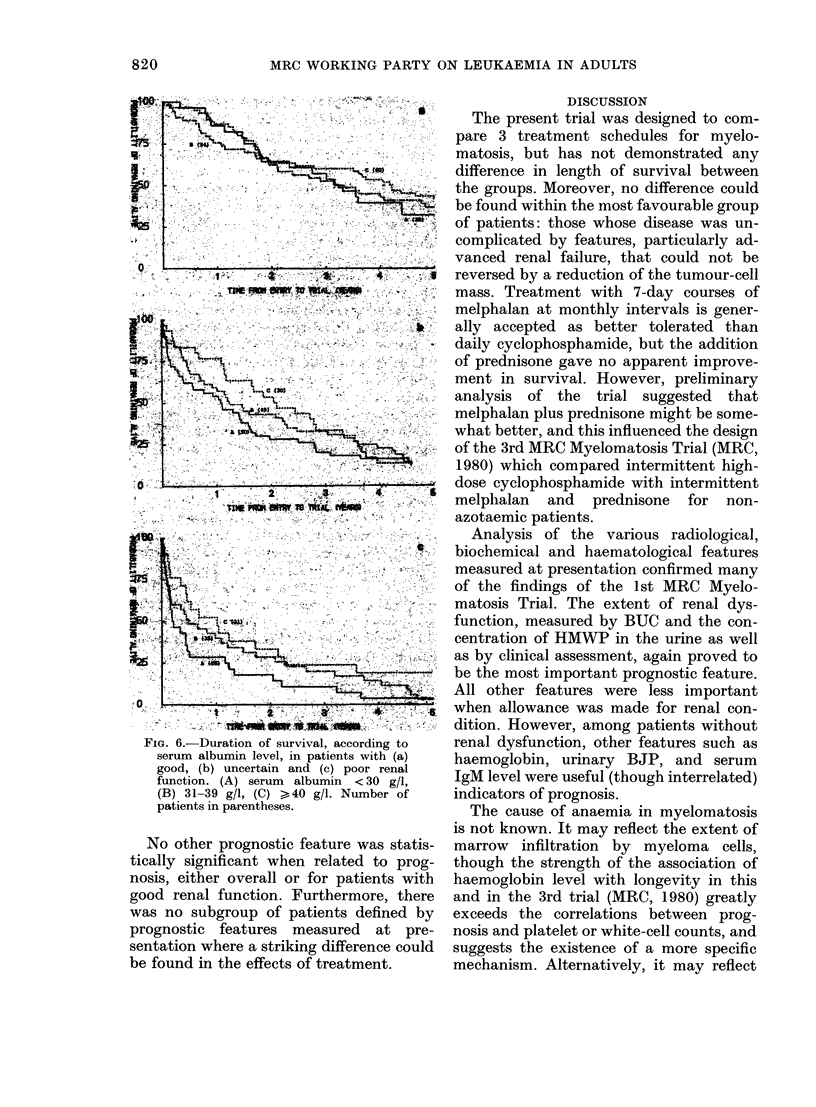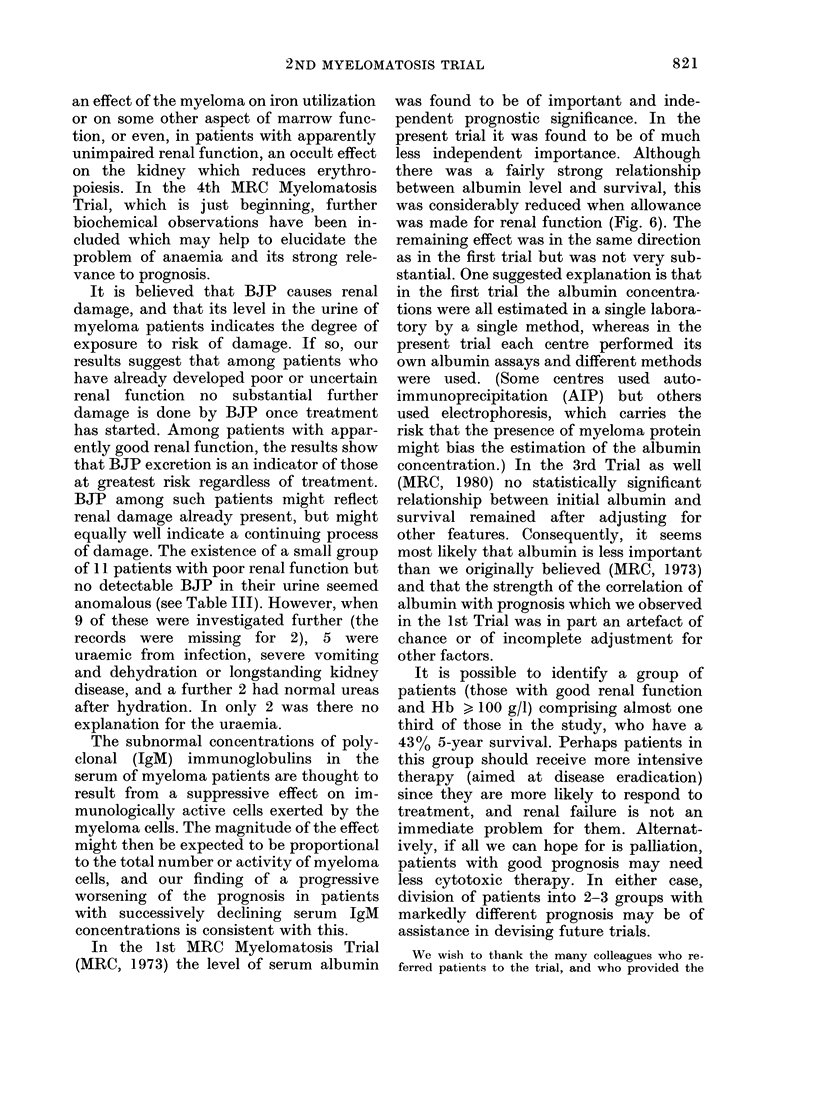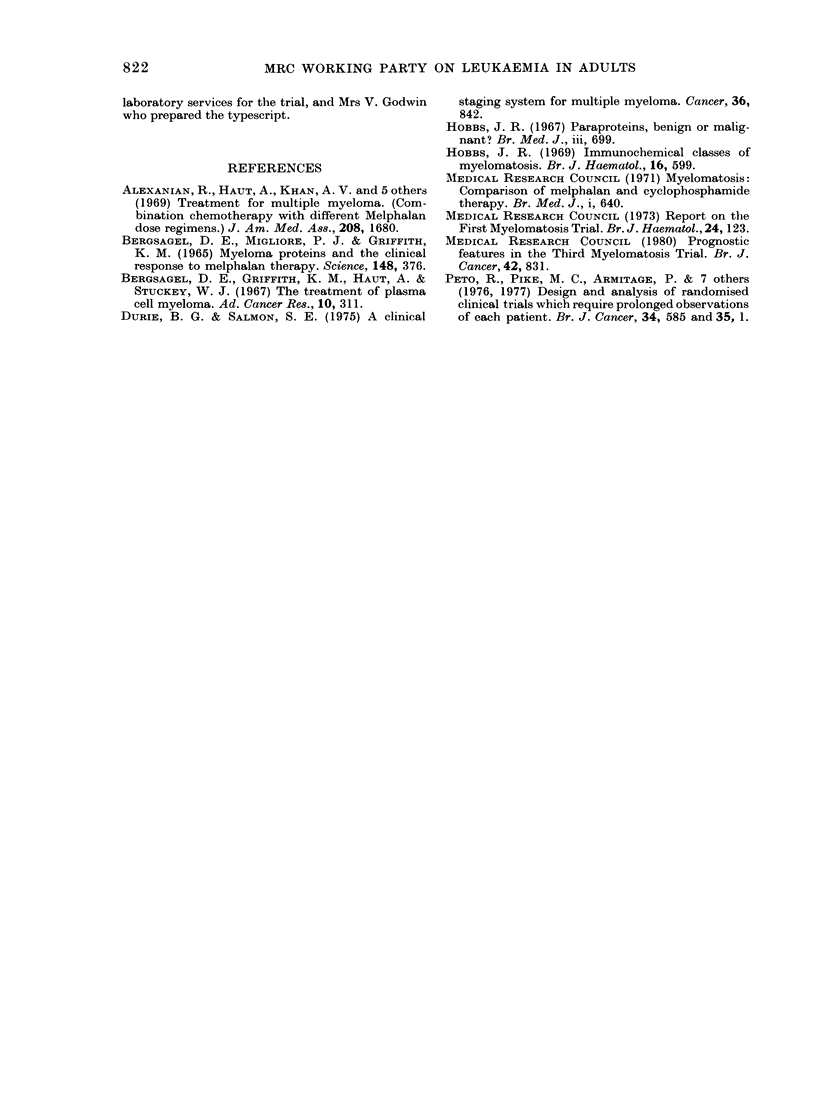# Report on the second myelomatosis trial after five years of follow-up. Medical Research Council's Working Party on Leukaemia in Adults.

**DOI:** 10.1038/bjc.1980.328

**Published:** 1980-12

**Authors:** 

## Abstract

Three hundred and seventy-two patients were randomized between 3 regimens of chemotherapy: cyclophosphamide, intermittent melphalan, and melphalan with prednisone, and were followed up to death or for at least 5 years. There was no difference in survival between the treatments, either overall or in any subgroup of patients. Therefore, the choice among these 3 treatments should be guided by the patient's comfort and convenience. The most important prognostic feature at presentation was the quality of renal function. It was possible to define good, intermediate and poor renal-function groups which were highly correlated with prognosis (X2 for trend = 62.6). The haemoglobin level at presentation was strongly correlated with prognosis among patients in the good renal-function group. Among 107 patients who presented with good renal function and with haemoglobin above 100 g/l, the 5-year survival was 43%. Other prognostic features were much less important when account was taken of renal function and haemoglobin level.


Br. J. Cancer (1980) 42, 813

### REPORT ON THE SECOND MYELOMATOSIS TRIAL AFTER FIVE YEARS OF FOLLOW-UP

MEDICAL RESEARCH COUNCIL'S
WORKING PARTY ON LEUKAEMIA IN ADULTS

The members of the Working Party over the period of the trial were Sir John Dacie (Chairman), D. A. G. Galton (Secretary), K. D. Bagshawe, P. Barkhan, A. J. Bellingham, E. K. Blackburn, S. Callender, I. W. Delamore, Sir Richard Doll, J. Durrant, J. J. Fennelly, I. D. Fraser, F. J. G. Hayhoe, J. R. Hobbs, J. Innes, H. E. M. Kay, G. W. Marsh, G. A. McDonald, I. C. M. MacLennan, M. G. Nelson, R. Peto, R. Powles, O. S. Roath, B. E. Roberts, J. Stuart, R. B. Thompson, G. Wetherley-Mein, J. A. Whittaker and E. Wiltshaw.

This report was prepared by H. Cuckle, D. A. G. Galton, R. Peto, E. Paul and M. Gilham.

Received and accepted 18 August 1980

**Summary.—Three hundred and seventy-two patients were randomized between 3 regimens of chemotherapy: cyclophosphamide, intermittent melphalan, and melphalan with prednisone, and were followed up to death or for at least 5 years. There was no difference in survival between the treatments, either overall or in any subgroup of patients. Therefore, the choice among these 3 treatments should be guided by the patient's comfort and convenience.**

**The most important prognostic feature at presentation was the quality of renal function. It was possible to define good, intermediate and poor renal-function groups which were highly correlated with prognosis ($\chi^2$ for trend = 62·6). The haemoglobin level at presentation was strongly correlated with prognosis among patients in the good renal-function group. Among 107 patients who presented with good renal function and with haemoglobin above 100 g/l, the 5-year survival was 43%. Other prognostic features were much less important when account was taken of renal function and haemoglobin level.**

In the Medical Research Council's First Myelomatosis Trial, previously untreated patients were randomly allocated to receive daily oral cyclophosphamide or melphalan. By August 1968, when 276 patients had been entered into the first trial, there was no significant difference in survival between the two groups (MRC, 1971). During the course of that trial it was claimed by others that intermittent melphalan was more effective than continuous administration, and that the addition of prednisone to intermittent melphalan gave further benefit (Bergsagel *et al.*, 1967; Alexanian *et al.*, 1969). The MRC's Second Myelomatosis Trial, which we now report, was therefore started to compare cyclophosphamide at the same dosage as in the first trial with a schedule of intermittent melphalan, and intermittent melphalan with prednisone.

*The protocols*

*Cyclophosphamide* was administered orally at a daily dose of 150 mg or, if neutropenia or thrombocytopenia developed (see below) at the highest tolerated dose.

*Intermittent melphalan* was administered in a short 4-day course and subsequent 7-day courses. First course: 10 mg daily for 4 days. If the blood urea concentration (BUC) was over 13·3 mM, treatment was delayed until the patient had been adequately hydrated, and then 5 mg was given daily for 4 days. Second and subsequent courses: 10 mg daily for 7 days or 5 mg daily for 7 days if BUC still exceeded 13·3 mM, reduced to 3–5 days if it considerably exceeded 13·3 mM.

The interval between courses was usually 6–8 weeks, depending on the trends in the neutrophil and platelet counts. Treatment was deferred or reduced if the neutrophil count remained below $2{\cdot}5 \times 10^9$/l and/or the platelet count remained below $100 \times 10^9$/l.

*Intermittent melphalan and prednisone.*—Melphalan was administered intermittently as above, each daily dose being accompanied by 40 mg of prednisone. After the end of each course of melphalan, 30, 20, 15, 10 and 5 mg of prednisone were given on successive days.

On all 3 regimens, radiotherapy (except for relief of pain), androgens, oestrogens and anabolic steroids as supportive therapy were to be avoided unless chemotherapy alone proved inadequate. Blood transfusions and analgesics could be used as required.

### *Eligibility for entry to the trial*

Patients with myelomatosis were not considered eligible unless abnormal plasma cells had been identified in marrow films or sections and, in addition, characteristic skeletal lesions had been demonstrated radiologically, and/or characteristic protein changes had been found in the serum or urine. Patients who had previously received urethane or cytotoxic treatment were ineligible. Previous *local* irradiation of a single painful site, or temporary irradiation for not more than 3 weeks, or administration of adrenal corticosteroids for the treatment of acute hypercalcaemia, or previous administration of androgens, oestrogens or anabolic steroids, did not preclude admission to the trial. Eligible patients were randomized to one of the 3 treatment schedules by telephone to the Leukaemia Trials Office in London.

### STATISTICAL METHODS

These are as described in the report on statistical methods to the Medical Research Council's Leukaemia Steering Committee (Peto *et al.*, 1976, 1977). They chiefly involve the plotting of Kaplan-Meier life-table estimates of the percentages of patients alive at various times up to 5 years after entry to illustrate various patterns of survival, and the calculation of logrank *P*-values to test the statistical significance of any apparent differences in survival. For the latter, exact variance calculations were made (*ibid.*, Statistical Note 7) and continuity corrections were not used (*ibid.*, p. 38). Chi-square values without subscripts are for the trend statistic unless otherwise stated, and therefore have only one degree of freedom.

### DATA COLLECTED

Between September 1968 and May 1975, 383 patients from 19 centres were randomized by telephone, but for 11 of these, repeated subsequent requests for data failed to locate a record of any such patient. Of the remaining 372, 124 were allocated to receive cyclophosphamide, 128 melphalan alone and 120 melphalan with prednisone. All but 2 patients were followed up to death or for at least 5 years (until 31 December 1979). These 2 were last known to be alive 3 and 21 months after entry respectively, and it has not been possible to trace them further. Whenever a patient died, the date of death was recorded and the cause of death sought.

The following information was requested on presentation: sex, age, serum albumin, calcium, IgM, alkaline phosphatase, BUC and haemoglobin levels, platelet and leucocyte counts, and serum paraprotein levels. Urine was collected for paraprotein type (heavy and light chain) and the concentrations of Bence-Jones protein (BJP) and high-mol.-wt proteins (HMWP, of higher molecular weight than BJP). Radiological screening for lytic lesions, pathological fractures or vertebral wedging was also requested. Information on whether the BUC related to a pre- or post-hydration sample was not specifically sought. During the first few weeks of therapy a clinical assessment of renal function was recorded in the following categories: "out of control", "no reason at all to suspect any impairment" or "other". Three months after randomization, the serum paraprotein level was again recorded.

TABLE I.—*Relative death rates according to blood urea concentration (BUC) and concentration of high-molecular-weight protein (HMWP) in the urine*

| BUC (mM) | HMWP (g/l) <0·1 | 0·1–0·99 | ≥1·0 | Total | $\chi^2$ for trend with respect to HMWP |
|---|---|---|---|---|---|
| <7·0 | 0·60 (81) | 0·80 (63) | 1·87 (17) | 0·73 | 12·16 ($P<0·001$) |
| 7·0–13·9 | 1·03 (38) | 1·33 (49) | 1·17 (31) | 1·18 | 0·25 (NS) |
| ≥14·0 | 1·86 (19) | 1·79 (31) | 1·65 (26) | 1·75 | 0·09 (NS) |
| Total | 0·80 | 1·07 | 1·42 | 1·00 | 14·69 ($P<0·001$) |

Numbers of patients in parentheses. The relative death rate for a cell of the table is the ratio of the observed number of deaths to that expected from the extent of exposure to risk of death experienced by patients in that cell.
NS = not significant.

The immunoglobulins in the serum and urine were estimated in one central laboratory by cellulose-acetate electrophoresis and typed by electrophoresis (Hobbs, 1967). The radiological findings were abstracted from local skeletal surveys of the skull, axial skeleton and long bones.

FIG. 1.—Duration of survival for patients randomized to receive cyclophosphamide (A), melphalan (B) or melphalan with prednisone (C). Numbers of patients in parantheses.

### RESULTS

In addition to the effects of the 3 treatments on survival, the various presentation features recorded for all 372 patients were considered in relation to prognosis. For those patients on whom the relevant data were available, other features also considered were the serum levels of albumin, IgM, paraprotein and calcium, the paraprotein type and urinary BJP and HMWP concentration.

### *Effect of treatment on survival*

There was no apparent difference in survival between the 3 treatment schedules ($\chi^2 = 0·09$, Fig. 1). Overall, the median length of survival was 20 months from the date of randomization.

### *Renal function*

The strongest determinants of prognosis in patients with myelomatosis have been shown to be those indicating renal impairment, namely elevated BUC and excessive amounts of HMWP in the urine. The former is an indicator of a reduced rate of glomerular filtration, and the latter of glomerular damage. Table I shows the relationship between these 2 indices and prognosis. Among patients whose blood urea was high (BUC ≥7·0 mM) the presence or absence of HMWP in the urine was of little or no prognostic significance. However, among patients whose blood urea was normal at presentation, those having HMWP in the urine faired considerably worse than those who did not ($\chi^2 = 12·16$, $P<0·001$). The blood urea and urinary proteins are objective laboratory measurements, but are subject to considerable uncertainty (not least because they were sometimes recorded before, and sometimes after, the hydration of the

FIG. 2.—Duration of survival for patients according to renal function at randomization (see text for the definition of these 3 categories). Numbers of patients in parentheses.

patient, and there is no reliable record of the timing). Therefore, we also asked the clinician to record at entry an impression of the renal condition (see above). Not surprisingly, this clinical impression was of statistically significant assistance in predicting mortality, even among patients who were still normal in terms of blood urea and urinary proteins. It was, therefore, possible to define a composite variable which more accurately reflected renal function in relation to survival by dividing patients into 3 groups:

(a) Good renal function; blood urea $<7{\cdot}0$ mM, $<1{\cdot}0$ g/l (if measured) of HMWP in the urine and "no reason at all to suspect any renal impairment" (145 patients).

(b) Uncertain renal function; those not in (a) or (c) (133 patients).

(c) Poor renal function; blood urea $\geqslant 14{\cdot}0$ mM or renal function described as "out of control" (94 patients).

The survival curves for these 3 renal-function groups are shown in Fig. 2 ($\chi^2 = 62{\cdot}6$). This 3-value composite variable will be used in the rest of the analyses to allow for the effect of renal impairment in the assessment of the other prognostic features.

*Anaemia*

The relationship between haemoglobin value at presentation and prognosis was

TABLE II.—*Relationship between renal function and haemoglobin concentration*

| Hb (g/l) | No. (%) whose renal function was: Good | Uncertain | Poor | Total |
|---|---|---|---|---|
| < 75 | 5 (9) | 28 (53) | 20 (38) | 53 |
| 75–99 | 33 (28) | 49 (42) | 36 (31) | 118 |
| 100+ | 107 (53) | 56 (28) | 38 (19) | 201 |
| Total | 145 (39) | 133 (36) | 94 (25) | 372 |

highly significant statistically ($\chi^2 = 21{\cdot}1$). Much of this was due to the strong correlation of haemoglobin concentration with renal function (Table II). Only 9% of patients with a low haemoglobin ($<75$ g/l) had good renal function, whereas 53% of those with a high haemoglobin ($\geqslant 100$ g/l) had no indication of renal damage. When allowance was made for renal function in the analysis, a definite effect of haemoglobin level on survival was present only in patients with good renal function ($\chi^2 = 7{\cdot}3$; $P < 0{\cdot}01$; see Fig. 3) and not at all in the other two renal-function categories ($\chi^2 = 0{\cdot}6$ and $0{\cdot}1$ respectively). There were 107 patients (29% of those in the study) who had both good renal function and haemoglobin level $\geqslant 100$ g/l. The median length of survival for these patients was 55 months.

In the third myeloma trial (MRC, 1980) it was found that clinical performance at presentation ("asymptomatic or minimal symptoms" versus "restricted activity or bedridden") was related to prognosis independently of BUC and haemoglobin.

FIG. 3.—Duration of survival for patients with good renal function according to their haemoglobin level. Numbers of patients in parentheses.

Information on initial clinical performance was not explicitly requested in the second trial, but it could be estimated for 330 of the 372 patients by reviewing their records. With this retrospective approach it was possible to confirm the importance of initial clinical performance as a predictor of prognosis, even among patients with apparently similar renal function and haemoglobin level ($\chi^2 = 9{\cdot}94$, $P = 0{\cdot}002$, for performance status restrospectively stratified for renal function groups and haemoglobin levels).

*Treatment among patients with normal haemoglobin (> 100 g/l) and good renal function*

Because of the overriding effect of impaired renal function on prognosis, any real benefits of a treatment which effectively reduced the total tumour-cell mass might be obscured by irreversible renal damage. The apparent lack of difference between the 3 treatments (as shown in Fig. 1) might have arisen because real but small differences between the 3 treatments were overshadowed by the effects of renal damage. To discover whether there were treatment differences among patients whose disease was uncomplicated by such features, we have therefore compared the results for the 107 patients with good renal function and haemoglobin level of ⩾100 g/l. However, there was still no evidence of any material difference between the 3 treatments (Fig. 4).

Fig. 4.—Duration of survival for patients with good renal function and haemoglobin level ⩾100 g/l, according to treatment: (A) cyclophosphamide, (B) melphalan and (C) melphalan with prednisone. Numbers of patients in parentheses.

*Bence Jones proteinuria (BJP)*

The presence of BJP was strongly correlated with poor prognosis ($\chi^2 = 27{\cdot}9$), but again this was in part due to the fact that heavy BJP was usually associated with impaired renal function. When the effects of BJP were examined within separate categories of patient, the only patients among whom it was prognostically important were those with good renal function and normal (⩾100 g/l) haemoglobin. The relationship of BJP to survival among patients with good renal function is illustrated in Fig. 5(a) ($\chi^2 = 13{\cdot}2$, $P < 0{\cdot}005$).

*M-immunoglobulin*

A very strong association was found between serum IgM level and prognosis

Fig. 5.—Duration of survival for patients with good renal function according to levels of (a) urinary BJP or (b) serum IgM. Numbers of patients in parentheses.

TABLE III.—*Survival in relation to various factors recorded at presentation, overall and within renal-function categories*

| | All patients | | | | | Renal function: Good | | | | | Renal function: Uncertain | | | | | Renal function: Poor | | | | |
|---|---|---|---|---|---|---|---|---|---|---|---|---|---|---|---|---|---|---|---|---|
| Factor | N | O | E | O/E | $\chi^2$ | N | O | E | O/E | $\chi^2$ | N | O | E | O/E | $\chi^2$ | N | O | E | O/E | $\chi^2$ |
| Hb (g/l) | | | | | | | | | | | | | | | | | | | | |
| <75 | 53 | 49 | 31·0 | 1·58 | | 5 | 3 | 3·0 | — | | 28 | 26 | 23·6 | 1·10 | | 20 | 20 | 14·2 | 1·40 | |
| 75–99 | 118 | 107 | 87·7 | 1·22 | 21·1 | 33 | 29 | 17·0 | 1·71 | 7·3 | 49 | 45 | 44·3 | 1·02 | 0·4 | 36 | 33 | 39·9 | 0·83 | 0·1 |
| 100+ | 201 | 176 | 213·3 | 0·83 | $P<0·0001$ | 107 | 86 | 98·0 | 0·88 | $P=0·007$ | 56 | 54 | 57·1 | 0·94 | NS | 38 | 36 | 34·9 | 1·03 | NS |
| BJP (g/l) | | | | | | | | | | | | | | | | | | | | |
| None | 113 | 89 | 127·7 | 0·70 | | 68 | 47 | 64·4 | 0·73 | | 34 | 31 | 37·4 | 0·83 | | 11 | 11 | 6·1 | 1·81 | |
| 0·01–0·99 | 121 | 114 | 109·7 | 1·04 | 27·4 | 45 | 41 | 34·4 | 1·19 | 13·2 | 46 | 45 | 43·5 | 1·03 | 1·5 | 30 | 28 | 37·4 | 0·75 | <0·01 |
| 1·0+ | 121 | 113 | 78·6 | 1·44 | $P<0·0001$ | 29 | 27 | 16·2 | 1·67 | $P=0·0003$ | 49 | 45 | 40·1 | 1·12 | NS | 43 | 41 | 36·5 | 1·12 | NS |
| IgM (g/l) | | | | | | | | | | | | | | | | | | | | |
| <0·15 | 98 | 95 | 67·1 | 1·41 | | 30 | 28 | 17·7 | 1·58 | | 37 | 36 | 27·8 | 1·29 | | 31 | 31 | 31·3 | 0·99 | |
| 0·15–0·29 | 98 | 94 | 83·7 | 1·12 | 26·3 | 46 | 45 | 32·1 | 1·40 | 20·2 | 31 | 29 | 27·7 | 1·05 | 4·6 | 21 | 20 | 19·6 | 1·02 | 0·01 |
| 0·30+ | 98 | 74 | 112·2 | 0·66 | $P<0·0001$ | 45 | 26 | 49·2 | 0·53 | $P<0·0001$ | 35 | 31 | 39·5 | 0·77 | $P=0·03$ | 18 | 17 | 17·1 | 1·00 | NS |
| Age | | | | | | | | | | | | | | | | | | | | |
| <60 | 106 | 86 | 121·5 | 0·71 | | 56 | 43 | 51·9 | 0·83 | | 31 | 27 | 35·5 | 0·76 | | 19 | 16 | 25·2 | 0·64 | |
| 60–69 | 145 | 130 | 135·0 | 0·96 | 31·0 | 63 | 53 | 50·6 | 1·05 | 4·1 | 49 | 45 | 51·5 | 0·87 | 7·4 | 33 | 32 | 26·5 | 1·21 | 2·4 |
| 70+ | 121 | 116 | 75·5 | 1·54 | $P<0·0001$ | 26 | 22 | 15·4 | 1·42 | $P=0·04$ | 53 | 53 | 38·0 | 1·39 | $P=0·006$ | 42 | 41 | 37·5 | 1·09 | NS |

N = Number of patients.

O = Observed number of deaths.

E = Extent of exposure to risk of death. For a group with average longevity E will approximately equal 0 (and O/E will be about 1) but for a group of patients who live for a very long time the extent of exposure to risk, E, will exceed 0 (and may even exceed N). The $\chi^2$ statistic compares the Os with the Es, and is a trend statistic based on one degree of freedom; $P$ denotes the probability associated with such a $\chi^2$ and NS indicates a non-significant value (*i.e.* $P>0·1$).

($\chi^2 = 26{\cdot}3$). This effect was clearly present in the good renal-function groups ($\chi^2 = 20{\cdot}2$); Fig. 5(b); less definitely present in the uncertain renal-function group ($\chi^2 = 4{\cdot}6$, $P < 0{\cdot}05$) and absent in those with poor renal function ($\chi^2 < 0{\cdot}01$). Among the 87 patients with good renal function and haemoglobin level $\geqslant 100$ g/l who had IgM measurements, the group of 33 with IgM > 0·30 g/l had a death rate 36% of that in the group with IgM concentration $\leqslant 0{\cdot}3$g/l.

*Age*

Younger patients had markedly better life expectancy ($\chi^2 = 31{\cdot}0$). This was because of poorer renal function in older patients, but within each of the 3 renal-function groups there was some residual effect of age ($\chi^2 = 4{\cdot}1$, 7·4 and 2·4) which is not surprising since the period of follow-up constitutes a substantial fraction of the remaining life expectancy of the older patients.

The effects of renal function on the relationship between prognosis and haemoglobin, BJP, IgM and age are summarized in Table III.

*Paraproteins*

The level of paraprotein in the serum (of patients without BJP myeloma) bore no relationship to prognosis for IgG patients, but among IgA patients there was a non-significant tendency for those with higher paraprotein concentrations to die earlier. The observation arising from the 1st MRC trial (1973) that patients whose serum paraprotein level fell slowly during treatment did better than those whose level decreased rapidly, was not confirmed. In the present trial, faster responders fared better than slow responders, and better than those who did not respond at all, though the differences were not statistically significant.

Of the 354 patients whose paraprotein type was recorded, 39 (11%) had BJP and no monoclonal heavy chain, 102 (29%) had IgA, 207 (58%) had IgG, and 6 (2%) had IgD. Overall there was no statistically significant correlation of type with survival, but among patients whose paraprotein level was raised ($\geqslant 40$ g/l for IgG and $\geqslant 30$ g/l for IgA) those with IgA had a worse prognosis than those with IgG ($\chi^2 = 3{\cdot}88$, $P < 0{\cdot}05$). Patients with only BJP presented on average with more BJP in their urine (4·46 g/l) than patients who also had IgA or IgG (1·15 g/l) and a slightly higher mean BUC (10·6 *vs* 10·1 mM). In contrast with the 1st MRC Myeloma Trial (Hobbs, 1969) the frequency of lytic lesions and hypercalcaemia was similar in all types of myeloma.

Fifty-three percent (185/352) of patients whose light-chain type could be determined had Type $\kappa$ light chains, but light-chain type was unrelated to prognosis. Within groups of patients of each heavy-chain type, those patients with Type $\lambda$ light chains had a higher urinary BJP concentration (overall mean 2·05 g/l compared with 1·05 g/l for Type $\kappa$ myelomas) and a higher BUC (11·95 mM compared with 8·63 mM for Type $\kappa$ myelomas). In contrast with the 1st MRC Trial patients with poor renal function who excreted Type $\lambda$ light chains did no better than those excreting Type $\kappa$ light chains. The suggestion that patients who excrete Type $\kappa$ respond better to melphalan than those who excrete Type $\lambda$ (Bergsagel *et al.*, 1965; Hobbs, 1969) was also not confirmed.

*Other features*

Platelet count and the levels of serum albumin and alkaline phosphatase at presentation were independently related to prognosis, to degrees which were statistically significant ($\chi^2 = 10{\cdot}7$, 12·9 and 3·9 respectively) but each effect was reduced when adjustment was made for renal function ($\chi^2 = 5{\cdot}8$, 5·5 and 3·1 respectively). The only factor which retained significance within a renal-function group was the level of serum albumin in patients with poor renal function. In all 3 renal-function groups, patients with low platelet counts tended to do worse than the others during the first 12 months of treatment, but thereafter their prognosis was similar.

Fig. 6.—Duration of survival, according to serum albumin level, in patients with (a) good, (b) uncertain and (c) poor renal function. (A) serum albumin $<30$ g/l, (B) 31–39 g/l, (C) $\geqslant 40$ g/l. Number of patients in parentheses.

No other prognostic feature was statistically significant when related to prognosis, either overall or for patients with good renal function. Furthermore, there was no subgroup of patients defined by prognostic features measured at presentation where a striking difference could be found in the effects of treatment.

### DISCUSSION

The present trial was designed to compare 3 treatment schedules for myelomatosis, but has not demonstrated any difference in length of survival between the groups. Moreover, no difference could be found within the most favourable group of patients: those whose disease was uncomplicated by features, particularly advanced renal failure, that could not be reversed by a reduction of the tumour-cell mass. Treatment with 7-day courses of melphalan at monthly intervals is generally accepted as better tolerated than daily cyclophosphamide, but the addition of prednisone gave no apparent improvement in survival. However, preliminary analysis of the trial suggested that melphalan plus prednisone might be somewhat better, and this influenced the design of the 3rd MRC Myelomatosis Trial (MRC, 1980) which compared intermittent high-dose cyclophosphamide with intermittent melphalan and prednisone for non-azotaemic patients.

Analysis of the various radiological, biochemical and haematological features measured at presentation confirmed many of the findings of the 1st MRC Myelomatosis Trial. The extent of renal dysfunction, measured by BUC and the concentration of HMWP in the urine as well as by clinical assessment, again proved to be the most important prognostic feature. All other features were less important when allowance was made for renal condition. However, among patients without renal dysfunction, other features such as haemoglobin, urinary BJP, and serum IgM level were useful (though interrelated) indicators of prognosis.

The cause of anaemia in myelomatosis is not known. It may reflect the extent of marrow infiltration by myeloma cells, though the strength of the association of haemoglobin level with longevity in this and in the 3rd trial (MRC, 1980) greatly exceeds the correlations between prognosis and platelet or white-cell counts, and suggests the existence of a more specific mechanism. Alternatively, it may reflect an effect of the myeloma on iron utilization or on some other aspect of marrow function, or even, in patients with apparently unimpaired renal function, an occult effect on the kidney which reduces erythropoiesis. In the 4th MRC Myelomatosis Trial, which is just beginning, further biochemical observations have been included which may help to elucidate the problem of anaemia and its strong relevance to prognosis.

It is believed that BJP causes renal damage, and that its level in the urine of myeloma patients indicates the degree of exposure to risk of damage. If so, our results suggest that among patients who have already developed poor or uncertain renal function no substantial further damage is done by BJP once treatment has started. Among patients with apparently good renal function, the results show that BJP excretion is an indicator of those at greatest risk regardless of treatment. BJP among such patients might reflect renal damage already present, but might equally well indicate a continuing process of damage. The existence of a small group of 11 patients with poor renal function but no detectable BJP in their urine seemed anomalous (see Table III). However, when 9 of these were investigated further (the records were missing for 2), 5 were uraemic from infection, severe vomiting and dehydration or longstanding kidney disease, and a further 2 had normal ureas after hydration. In only 2 was there no explanation for the uraemia.

The subnormal concentrations of polyclonal (IgM) immunoglobulins in the serum of myeloma patients are thought to result from a suppressive effect on immunologically active cells exerted by the myeloma cells. The magnitude of the effect might then be expected to be proportional to the total number or activity of myeloma cells, and our finding of a progressive worsening of the prognosis in patients with successively declining serum IgM concentrations is consistent with this.

In the 1st MRC Myelomatosis Trial (MRC, 1973) the level of serum albumin was found to be of important and independent prognostic significance. In the present trial it was found to be of much less independent importance. Although there was a fairly strong relationship between albumin level and survival, this was considerably reduced when allowance was made for renal function (Fig. 6). The remaining effect was in the same direction as in the first trial but was not very substantial. One suggested explanation is that in the first trial the albumin concentrations were all estimated in a single laboratory by a single method, whereas in the present trial each centre performed its own albumin assays and different methods were used. (Some centres used autoimmunoprecipitation (AIP) but others used electrophoresis, which carries the risk that the presence of myeloma protein might bias the estimation of the albumin concentration.) In the 3rd Trial as well (MRC, 1980) no statistically significant relationship between initial albumin and survival remained after adjusting for other features. Consequently, it seems most likely that albumin is less important than we originally believed (MRC, 1973) and that the strength of the correlation of albumin with prognosis which we observed in the 1st Trial was in part an artefact of chance or of incomplete adjustment for other factors.

It is possible to identify a group of patients (those with good renal function and Hb $\geqslant 100$ g/l) comprising almost one third of those in the study, who have a 43% 5-year survival. Perhaps patients in this group should receive more intensive therapy (aimed at disease eradication) since they are more likely to respond to treatment, and renal failure is not an immediate problem for them. Alternatively, if all we can hope for is palliation, patients with good prognosis may need less cytotoxic therapy. In either case, division of patients into 2–3 groups with markedly different prognosis may be of assistance in devising future trials.

We wish to thank the many colleagues who referred patients to the trial, and who provided the laboratory services for the trial, and Mrs V. Godwin who prepared the typescript.

### REFERENCES

ALEXANIAN, R., HAUT, A., KHAN, A. V. and 5 others (1969) Treatment for multiple myeloma. (Combination chemotherapy with different Melphalan dose regimens.) *J. Am. Med. Ass.*, **208**, 1680.

BERGSAGEL, D. E., MIGLIORE, P. J. & GRIFFITH, K. M. (1965) Myeloma proteins and the clinical response to melphalan therapy. *Science*, **148**, 376.

BERGSAGEL, D. E., GRIFFITH, K. M., HAUT, A. & STUCKEY, W. J. (1967) The treatment of plasma cell myeloma. *Ad. Cancer Res.*, **10**, 311.

DURIE, B. G. & SALMON, S. E. (1975) A clinical staging system for multiple myeloma. *Cancer*, **36**, 842.

HOBBS, J. R. (1967) Paraproteins, benign or malignant? *Br. Med. J.*, iii, 699.

HOBBS, J. R. (1969) Immunochemical classes of myelomatosis. *Br. J. Haematol.*, **16**, 599.

MEDICAL RESEARCH COUNCIL (1971) Myelomatosis: Comparison of melphalan and cyclophosphamide therapy. *Br. Med. J.*, i, 640.

MEDICAL RESEARCH COUNCIL (1973) Report on the First Myelomatosis Trial. *Br. J. Haematol.*, **24**, 123.

MEDICAL RESEARCH COUNCIL (1980) Prognostic features in the Third Myelomatosis Trial. *Br. J. Cancer*, **42**, 831.

PETO, R., PIKE, M. C., ARMITAGE, P. & 7 others (1976, 1977) Design and analysis of randomised clinical trials which require prolonged observations of each patient. *Br. J. Cancer*, **34**, 585 and **35**, 1.